# Plant- and Microalgae-Based Biotechnological Strategies for Affordable and Non-Invasive Delivery of Antidiabetic Peptides

**DOI:** 10.3390/pharmaceutics18020223

**Published:** 2026-02-10

**Authors:** Thibault Boscart, Alexandre Barras, Valérie Plaisance, Valérie Pawlowski, Emerson Giovanelli, Muriel Bardor, Christophe D’Hulst, Amar Abderrahmani

**Affiliations:** 1 Univ. Lille, CNRS, Centrale Lille, University Polytechnique Hauts-De-France, UMR 8520, IEMN, 59000 Lille, France; thibault.boscart.etu@univ-lille.fr (T.B.); alexandre.barras@univ-lille.fr (A.B.); valerie.abderrahmani@univ-lille.fr (V.P.); valerie.pawlowski@univ-lille.fr (V.P.); emerson.giovanelli@univ-lille.fr (E.G.); 2 Univ. Lille, CNRS, UMR 8576-UGSF-Unité de Glycobiologie Structurale et Fonctionnelle, 59000 Lille, France; 3 Université de Rouen Normandie (UNIROUEN), Normandie University, GlycoMEV UR 4358, SFR Normandie Végétal FED 4277, Innovation Chimie Carnot, IRIB, GDR CNRS Chemobiologie, 76000 Rouen, France; muriel.bardor@algabiologics.com; 4 ALGA BIOLOGICS, 67 rue du Bouvreuil, 76230 Isneauville, France

**Keywords:** plants, microalgae, polysaccharides, diabetes, nanoparticles, oral administration

## Abstract

The prevalence of diabetes and its worldwide co-morbidities is escalating. Therefore, the number of users of therapeutic peptides including insulin analogs and glucagon-like peptide 1 receptor agonists (GLP-1RAs), will unavoidably increase in the coming years. However, access to these two antidiabetic classes remains limited in some countries due to their high cost. Even when available, their long-term therapeutic efficiency is often compromised by challenges in sustained treatment adherence, mainly resulting from their mode of administration through repeated subcutaneous injections. This repeated invasive delivery not only affects patient comfort but also complicates long-term disease management and monitoring. Therefore, there is an urgent need to improve the accessibility, affordability, and long-term patient adherence to insulin and GLP-1RAs. In this review, we highlight as promising alternatives the potential of plants and microalgae to serve as host organisms, as well as the use of their polysaccharides as drug carriers, for the production of low-cost and non-invasive antidiabetic drugs.

## 1. Introduction

Approximately 10% of the global population lives with diabetes, affecting every country worldwide [1]. The number of people suffering from this disease is projected to rise to 783 million by 2045, impacting both high- and low-income countries alike [1]. Diabetes is defined as an acute and chronic blood hyperglycemia, which is often accompanied by hyperlipidemia and systemic inflammation, most particularly when worsened by postprandial glucose spikes. These conditions expose patients to a significant risk of serious complications, including cardiovascular disease, neuropathy, retinopathy, nephropathy, and increased susceptibility to infections, often culminating in severe disabilities such as blindness or limb amputation [2]. On average, diabetes reduces life expectancy by six years and causes 6.7 million adult deaths globally in 2021 (~12.2% of deaths) [3]. There are four types of diabetes based on etiopathogenesis [4]. Aside from gestational diabetes (GD) and less common forms such as iatrogenic diabetes, exocrine pancreas-related diabetes (e.g., cystic fibrosis), and virus-induced diabetes, type 1 (T1D) and type 2 diabetes (T2D) account for over 90% of all diagnosed cases [5]. The standard treatment for T1D involves lifestyle changes alongside insulin administration using prefilled injection pens. However, fear of needles and injection discomfort can negatively impact patient adherence to the treatment over time, in spite of actual technological advances. These include painless needles or microneedle devices [6], but remain limited in terms of availability and affordability. For patients requiring lifelong therapy, the psychological and logistical burdens of regular injection can result in treatment discontinuation or irregular use, ultimately compromising glycemic control and increasing the risk of complications [7]. Insulin therapy is required in 15% to 25% of individuals with T2D, depending on the country, and not only when glycemic targets are not met despite the use of oral antidiabetic drugs (OADs) [8,9,10,11], but also as a consequence of the progressive nature of T2D. Over time, declining β-cell function reduces endogenous insulin secretion, making insulin initiation a physiologically expected step in long-term disease management rather than a limitation of OADs [10,12]. OADs remain central to T2D therapy, and ongoing advances, including optimized combination strategies, improved incretin-based mechanisms, and formulations aimed at supporting β-cell health, continue to strengthen their therapeutic role. However, the number of untreated or inadequately treated patients is expected to grow, particularly in low-resource areas, largely due to the limited accessibility and affordability of insulin. In addition to insulin, glucagon-like peptide-1 receptor agonists (GLP-1RAs) have emerged as a promising class of drugs for the treatment of T2D, particularly in overweight or obese patients. These agents not only improve glycemic control but also promote weight loss and may protect pancreatic β-cells from glucolipotoxicity and inflammatory stress [13,14]. Nevertheless, GLP-1RAs are expensive and, like insulin, are mostly administered via subcutaneous injection, posing similar limitations to patient adherence and accessibility [15,16]. Given the rising global burden of diabetes and the limitations of current injectable peptide therapies, there is an urgent need to develop innovative, cost-effective, and patient-friendly delivery methods for antidiabetic peptides. In this context, plants and microalgae have emerged as promising platforms for the production of therapeutic proteins and peptides. Their ability to serve as scalable, low-cost, and safe biofactories [17,18,19,20], combined with the potential of plant-derived or microalgae-derived polysaccharides for oral drug delivery, opens new perspectives to address unreached clinical needs in the management of diabetes. This narrative review aims to explore the dual role of plants and microalgae as platforms for the production of next-generation antidiabetic peptides, such as insulin and GLP-1RAs, and their associated polysaccharides for their oral delivery. By examining recent advances and ongoing challenges, we highlight the potential of green biotechnology to transform diabetes treatment and expand access to essential therapies worldwide.

## 2. Insulin and Glp-1 Receptor Agonists: Therapeutic Use and Accessibility

Despite major therapeutic advances over recent decades, the management of diabetes, particularly when reliant on injectable peptides such as insulin and GLP-1RAs, remains suboptimal in many populations. Multiple interrelated barriers continue to hinder effective, equitable, and sustained glycemic control across both T1D and T2D. Insulin remains the cornerstone therapy for T1D and for a growing subset of T2D patients for whom OADs fail to achieve glycemic targets. For this purpose, different types of insulin are used based on their short- or long-time action (Table 1). In T1D, the current treatment consists of the injection of long-acting insulin referred to as basal insulin, which is followed by the administration of short-acting insulin as the insulin bolus in the period of meal. In T2D, basal insulin is the current strategy, which is sometimes accompanied by the inclusion of short/rapid or intermediate-acting insulin in case the glycemic control is not achieved. However, despite the clinical effectiveness of insulin in diabetes management, its accessibility remains a major challenge. Indeed, the production of insulin involves high costs, contributing to significant disparities in treatment access.

Although recombinant DNA technology has enabled large-scale production of insulin, primarily using *Escherichia coli* or *Saccharomyces cerevisiae* as expression hosts, the manufacture of any type of insulin remains expensive due to complex purification steps, including the removal of cell-derived contaminants such as bacterial endotoxins [21,22]. Moreover, final pricing is influenced by government agencies and insurance systems, leading to stark disparities across countries [23,24]. The ACCISS (Addressing the Challenge and Constraints of Insulin Sources and Supply) study highlighted these disparities, revealing median prices ranging from $3.10 (median from $1.50 to $7.10) in upper-middle-income countries to $6.90 in low-income countries between 1996 and 2013 [23,24]. In contrast, prices ranged from $3.20 to $18.10 (median $4.00) in high-income countries over the same period. In the United States, up to 30% of diabetic patients report skipping insulin doses entirely due to cost, despite insurance coverage [25,26].

GLP-1RAs have recently emerged as highly effective antidiabetic agents in T2D that offer additional benefits in weight reduction, cardiovascular protection, and potential β-cell preservation [13,14]. There are various types of GLP-1RAs available in the market that mainly differ from each other depending on their half-life in blood and effects on weight loss (Table 2). However, their clinical utility remains limited by high costs and the fact that today, most formulations are mostly injectable. The pricing of GLP-1RAs often equals or exceeds that of insulin, particularly for long-acting analogs, making them inaccessible for many patients in low- and middle-income countries [15]. Moreover, actual adherence to GLP-1RAs therapies remains suboptimal, in part due to needle aversion, injection site reactions, and the required injection frequency, ranging from once daily to once weekly, as well as the long duration of treatment, which can be burdensome for many patients [16].

## 3. Current Challenges for Oral Delivery of Insulin and Glp-1ras

### 3.1. Overcoming Digestion of Peptides in Oral Delivery

Oral delivery represents a pinnacle of patient-centered therapy, removing both the physical and psychological burdens of injections and thereby enhancing adherence and concrete outcomes. Advances in formulation science now allow oral peptides to exhibit meaningful bioavailability, positioning oral delivery as a scalable and highly desirable alternative to injectable treatments. The principal obstacle remains the gastrointestinal tract (GIT) itself: acidic conditions and coordinated proteolytic activity both in the stomach and intestine are designed, from an evolutionary perspective, to digest dietary proteins. To address this, researchers have developed multiple strategies, including the use of protease inhibitors and nanoparticle-based delivery systems, ranging from synthetic polymers and lipids to inorganic nanoparticles, to enhance peptide stability and residence time in the gut, more specifically, for molecules such as insulin and GLP-1RAs [27].

Protease inhibitors, including aprotinin, soybean trypsin inhibitor, chicken egg inhibitors, and FK-448 chymotrypsin inhibitor, can shield peptides from enzymatic degradation, facilitating their absorption and improving their bioavailability [28,29,30,31]. However, chronic or systemic absorption of these inhibitors can disrupt normal digestion, cause systemic toxicity, or induce pancreatic hyperplasia [32,33], which limits their suitability for long-term therapy and raises safety and regulatory concerns. Nanoparticle-based carriers offer another kind of protection, relying on peptide encapsulation, protecting them from low pH and enzymatic degradation. Biodegradable polymers such as poly lactic-*co*-glycolic acid, chitosan, and hybrid lipid/polymer compositions have demonstrated protection of insulin and improved absorption in preclinical models [32,33,34,35]. Lipid-based nanocapsules, in particular, have stabilized GLP-1RAs in the gastrointestinal environment and promoted their uptake in proof-of-concept preclinical studies [36,37]. Despite these promising advances, clinical translation remains challenging due to manufacturing complexity, difficulties in reproducing particle size and surface chemistry in a perfectly controlled manner, and concerns regarding long-term accumulation or immunogenicity of synthetic nanomaterials [38]. Achieving efficient oral insulin and GLP-1RAs delivery thus requires the rational engineering of advanced carrier systems that remain inert during GIT, enabling controlled release and efficient absorption of therapeutic peptides.

### 3.2. Enabling Controlled Release and Intestinal Absorption of Peptides

**Figure 1:**
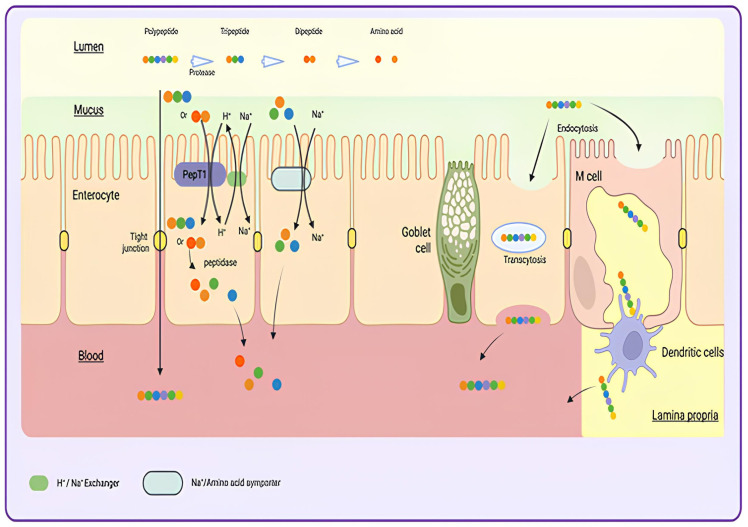
***Proposed mechanisms for the transport of peptides from the intestinal lumen into the blood circulation***. Unlike free amino acids, which are absorbed via specific Na^+^-dependent or Na^+^-independent transporters, small di- and tri-peptides are predominantly taken up by the proton-coupled oligopeptide transporter PepT1. Intact polypeptides can cross the intestinal epithelium through several alternative routes, including transcytosis via enterocytes and paracellular transport through tight-junction modulation, allowing limited passage of larger peptides (up to ~10 amino acids, ~1 kDa) between epithelial cells. Polypeptides can also cross through goblet cell-associated transport facilitated by mucus secretion, and M-cell-mediated uptake in Peyer’s patches, facilitating antigen sampling and translocation into underlying lymphoid tissue. Once past the epithelial barrier, polypeptides enter the lamina propria, diffuse into capillaries or lymphatic vessels, and ultimately reach systemic circulation. Goblet cells do not transport peptides directly; they facilitate absorption through their mucus secretion, which protects the epithelium and enhances peptide interaction with enterocytes for efficient uptake. The figure was created in Biorender. Boscart, T. (2026) https://Biorender.com/p5l62bp.

Together with protection from gastrointestinal degradation, efficient intestinal absorption of GLP-1RAs and insulin represents a key challenge. Free amino acids and small peptides are readily absorbed via dedicated transport systems in enterocytes [39], whereas larger proteins and polypeptides are mostly absorbed after enzymatic digestion, with over 90% taken up as free amino acids and less than 10% as small peptides. Only minimal amounts are absorbed as intact proteins or oligopeptides [40]. Notably, small peptides are absorbed faster than free amino acids, benefiting from preferential transport mechanisms [41]. Emerging evidence, however, suggests that intact oligopeptides and proteins can traverse the intestinal epithelium under physiological conditions, implying that enzymatic digestion is sometimes incomplete or locally bypassed. As a representative example, in rats infused with tritium-labeled bovine serum albumin (^3^H-BSA), approximately 2% of the full-length protein was detected in lymph and blood [42]. Similarly, other studies demonstrated the intestinal absorption of intact oligopeptides, including thyrotropin-releasing hormone (TRH), luteinizing hormone-releasing hormone (LHRH), and insulin [43]. Once across the epithelium, peptides enter the *lamina propria* and are transported via capillary or lymphatic vessels into systemic circulation (Figure 1). Even so, only a small fraction reaches the bloodstream [43]. In humans, evidence for intact protein and oligopeptide absorption remains more controversial [40]. In vitro studies using Caco-2 cell models of the human intestinal epithelium further highlight that transepithelial transport of intact oligopeptides is influenced by their susceptibility to enzymatic degradation. For example, bradykinin and glycine-glycine-tyrosine-arginine showed more efficient transport than β-casomorphin and ovokinin, due to reduced hydrolysis [44]. These findings support the concept that a significant fraction of intact oligopeptides can enter circulation if digestion is sufficiently limited [45].

Permeation enhancers provide another strategy to improve intestinal peptide absorption by transiently modulating tight junctions or membrane fluidity. Medium-chain fatty acids and surfactants, such as sodium caprate, have been shown to enhance insulin uptake [46,47,48]. The clinical potential of this approach is exemplified by oral semaglutide (Rybelsus^®^), the only oral GLP-1RA with Wegovy now currently approved. Incorporation of sodium N-(8 [2-hydroxybenzoyl]amino) caprylate (SNAC) with semaglutide protects the peptide from degradation while enabling transcellular intestinal uptake [49]. While permeation enhancers can significantly improve bioavailability, unfortunately, their chronic use raises safety concerns, including mucosal irritation and potential disruption of epithelial integrity, especially in vulnerable diabetic populations [50]. Furthermore, variability in enhancer effects along the gastrointestinal tract can compromise consistent dosing. Consequently, the next frontier in oral peptide therapeutics lies in designing innovative delivery systems that safely and reproducibly enable controlled intestinal release and absorption of both GLP-1RAs and insulin.

## 4. Plant- and Microalgae-Based Expression Systems and Bioencapsulation for Oral Peptide Delivery

### 4.1. Plant-Based Expression Platforms and Bioencapsulation for Oral Delivery

Plants have long been considered as potential carriers of proteins and peptides for oral delivery [51]. Such consideration relies on their cell wall, which is highly resistant to human enzymes. Other than pectin, small amounts of glycoproteins, phenolic acids, and minerals, the plant cell wall is composed of polysaccharides, including cellulose microfibrils, hemicelluloses, and lignin, which form rigid tubular structures [52]. This cellulose-hemicellulose-lignin biocomposite provides structural resistance, protecting plant cells from mechanical stress and environmental challenges [53]. Humans lack enzymes such as cellulase and ligninase, making the plant cell wall undigestible in the mouth, stomach, and intestine. Only starch-like plant polymers can be readily broken down by human amylase and maltase. However, thermal treatment (e.g., boiling, drying, baking, and roasting) and mechanical processes such as grinding and milling help disrupt the plant tissue matrix and break down cross-links in the cell wall, making it, as well as the cell contents, available for digestion [52]. In addition, gut bacteria can hydrolyze cellulose and lignin during the fermentation process in the large intestine. Therefore, in the absence of extensive processing, peptides produced in plants remain bioencapsulated within intact plant cells, where rigid cell walls and intracellular compartments protect them from degradation in the GIT.

**Figure 2:**
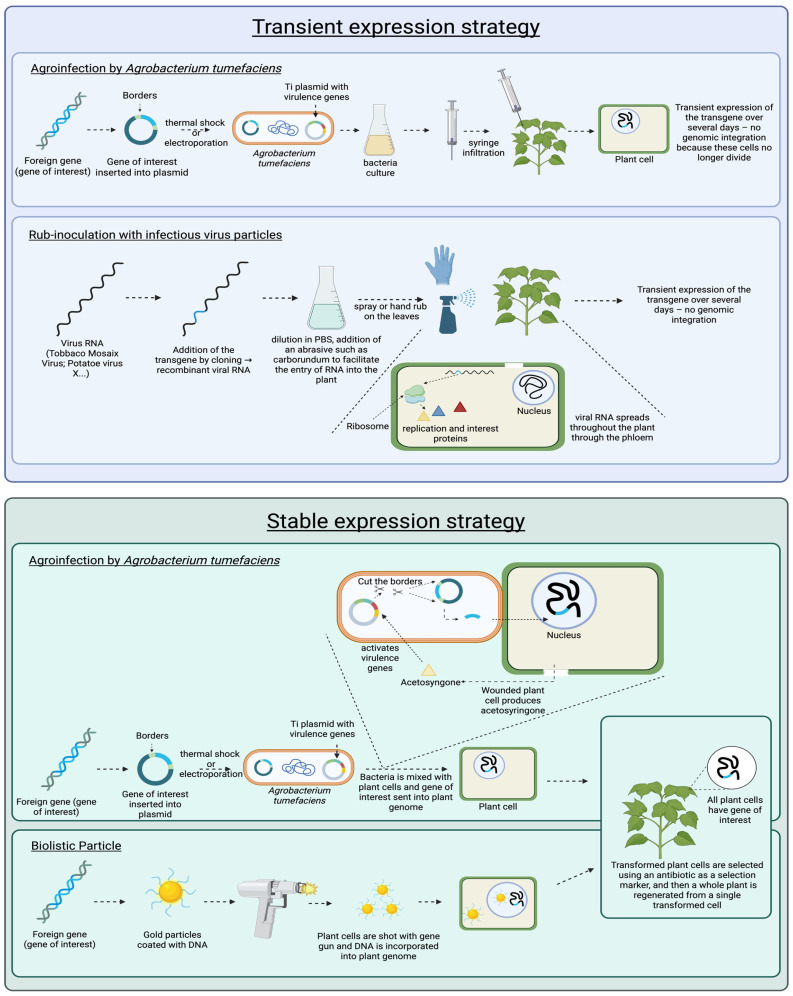
***Strategies for recombinant peptides expression in plants***. Transient expression allows short-term, high-level protein production without genomic integration, using approaches such as agroinfiltration or viral vector-mediated systems, frequently in *Nicotiana* species (top panel). Stable transformation involves the integration of foreign genes into the nuclear or plastid genome, generating stable transgenic lines that maintain therapeutic peptide expression across generations, typically via *Agrobacterium tumefaciens*-mediated transformation or biolistic delivery (bottom panel). The figure was created in Biorender. Boscart, T. (2026) https://Biorender.com/p5l62bp.

This natural bioencapsulation is achieved via plant molecular farming (PMF), whereby recombinant peptides are synthesized directly inside plant cells. These foreign peptides can be expressed either transiently for short-term high-level production or through stable transformation, allowing permanent expression across plant generations (Figure 2). Transient expression systems, most commonly agroinfiltration or viral vector-mediated expression in *Nicotiana* species, can yield recombinant proteins in the range of 50–500 mg/kg fresh weight, and in some optimized cases approaching 1 g/kg in some optimized cases. Stable nuclear transformation typically results in lower yields, often 1–50 mg/kg fresh weight, whereas chloroplast transformation can reach substantially higher levels, up to 1–3 g/kg fresh weight, and in extreme cases up to 10–70% of total soluble leaf protein for selected targets [54]. These yields are certainly generally lower and more variable than those achieved in microbial systems such as *E. coli* or yeast, where insulin and GLP-1RAs are routinely produced at 1–5 g/L culture volume under good manufacturing practice (GMP) conditions. But PMF offers instead advantages in protein quality, safety, and formulation flexibility. Importantly, plant cell walls function not only as protective barriers against digestive enzymes but also as intrinsic encapsulation matrices that can be directly leveraged for oral peptide delivery. Plant-based expression platforms offer several compelling advantages over traditional microbial cell-based systems (Table 3).

#### 4.1.1. Plant-Based Expression Systems for Insulin

Plant-based expression systems are currently used to produce some commercially available mammalian antibodies, blood substitutes, vaccines, and other therapeutic recombinant proteins for different applications and markets (Table 4) [55,56,57]. The first biological product obtained from such a system was glucocerebrosidase, approved in 2012 for enzyme replacement therapy of Gaucher’s disease [58]. Indeed, PMF offers the advantage of producing recombinant proteins in larger quantities than those produced using mammalian cell systems while avoiding contamination by human or animal pathogens [59]. Insulin is particularly well-suited to plant-based production because it is a non-glycosylated peptide hormone. Chloroplast transformation systems, which do not perform N-glycosylation but allow very high expression due to polyploidy and maternal inheritance, have been especially effective at producing insulin. Expression levels in chloroplasts can reach up to 70% of total leaf protein in optimized systems [54].

In plants, recombinant proteins can be expressed either from the nuclear genome or through plastid (chloroplast) transformation. Nuclear expression allows proper post-translational modification, including N-glycosylation, which is essential for the production of many biological products, as over 70% of them are glycoproteins. In contrast, chloroplast genomes do not perform N-glycosylation but allow very high expression levels, which can reach up to 70% of the total leaf protein [60], due to their high genome copy number and material inheritance, limiting transgene dissemination. Because insulin is a non-glycosylated peptide hormone, its production is particularly compatible with chloroplast expression systems of various plant species (Table 5), including transgenic *Arabidopsis thalania* (thale cress) [61], *Nicotiana benthamiana* (tobacco) [54,62], and *Lactuca sativa* (lettuce) [54,63]. One of the major examples was the recombinant human insulin produced by lettuce and entrapped into the chloroplasts [63]. While absolute oral bioavailability remained low, typically less than 1–2%, this represented still a 5 to 20-fold improvement compared with unprotected oral insulin. Oral administration of insulin-containing lettuce extracts into streptozotocin-induced diabetic mice significantly lowered glycemia [63]. In addition, it was observed that the glucose-lowering effect was less rapid, even if more prolonged than commercial insulin injection [63], suggesting that the oral insulin formulation is safer than injection regarding the hypoglycemia risk.

To improve the oral absorption and prevent the degradation caused by the gastrointestinal passage, insulin can be fused with several tags, including transferrin, protein transduction domain (PTD), dendritic cell peptide (DC-Pep), and cholera toxin B (CTB). These tags further strengthen the resistance of the recombinant peptides or proteins against digestion within the GIT, thereby improving the oral insulin bioavailability [51]. For example, transferrin is resistant to proteolytic enzymes, resulting in a prolonged half-life in blood (14–17 days) [73]. CTB has been shown to make some plant-produced polypeptide recombinants more resistant to digestive enzymes for oral delivery. That evidence has been first provided using tobacco leaves (*Nicotiana tabacum*) as the host to produce green fluorescent protein (GFP) fused with pentameric CTB [74]. After administration to mice by gavage, the CTB-GFP fusion protein has been found in spleen, intestinal mucosa, and hepatocytes, indicating that the intact CTB-GFP protein crosses the intestinal lumen [74]. As another example, CTB-insulin fusion protein produced by potato (*Solanum tuberosum*) improves the immunologic tolerance of non-obese diabetic (NOD) mice model of T1D [75]. NOD mice fed with CTB-insulin potato tuber developed serum and intestinal anti-CTB and anti-insulin IgG antibodies, suggesting that intact CTB-insulin fusion protein reaches the gut-associated lymphoid tissues. In such mice fed once per week for 5 weeks with CTB-insulin potato tuber, a decrease in the insulitis and a major delay in the onset of hyperglycemia were observed, confirming the oral immune tolerance achieved by CTB-insulin in the gut [74]. Improvement of immunological response and insulin transport across the gut by oral delivery of CTB-insulin in NOD mice has been later confirmed by other groups [76,77]. The successful production of bioactive insulin in plants demonstrates both the feasibility of PMF in the production of therapeutic peptides and the efficiency of such plant-based peptides as OADs. Of course, this approach can be extended to other peptide classes such as GLP-1RAs.

#### 4.1.2. Plant-Based Expression Systems for Glp-1ras

Plant-based production of GLP-1RAs fused with tags having antidiabetic effects has been reported in several studies (Table 5). Oral delivery of GLP-1RAs exendin 4 fused with CTB [69] or transferrin [72], produced, respectively, by *Lactuca sativa* and *Nicotiana benthamiana* chloroplasts, improves glucose tolerance in mice during an intraperitoneal glucose tolerance test. The use of plant expression systems could thus represent a novel and sustainable approach to expand the availability of GLP-1RAs, potentially transforming the landscape of metabolic disease therapeutics by improving accessibility and compliance through oral delivery mechanisms.

#### 4.1.3. Innovative Oral Delivery Systems of Insulin and Glp-1ras Using Plant Polysaccharides (Ps)-Based Nanoparticles

Beyond direct in planta production and bioencapsulation approaches, plant polysaccharides (PS) offer an outstanding opportunity for the development of carrier systems, particularly nanoparticles, for the oral delivery of insulin and GLP-1RAs. Unlike in planta expression strategies, which are constrained by variable expression levels, downstream processing, and limited control over dose uniformity, PS-based nanoparticles allow precise tuning of composition, size, and release kinetics. In contrast to bioencapsulation within plant tissues, which relies largely on passive protection mechanisms, nanoscale carriers can be rationally engineered to actively modulate peptide stability, intestinal permeability, and site-specific release. By combining biocompatibility, biodegradability, and inherent resistance to gastrointestinal conditions, naturally sourced PS enables the design of robust nanosystems capable of protecting peptides such as insulin and GLP-1RAs during gastric transit while promoting controlled and reproducible intestinal release. Through electrostatic interactions, hydrogen bonding, or gel network formation, numerous plant-derived PS nanoparticles can effectively encapsulate peptides, shielding them from enzymatic degradation and acidic pH during gastric transit, while maintaining structural integrity and enabling adhesion to the intestinal mucosa. PS nanoparticles (NPs) used for the delivery of insulin and GLP-1RAs include pectin, cellulose derivatives, and arabinoxylans (Table 6). These materials can form hydrogels or polyelectrolyte complexes that respond to pH changes in the GIT, allowing for targeted release in the intestine while remaining stable in acidic gastric conditions [78]. Chitosan, a cationic polymer, has been extensively studied for its mucoadhesive properties and ability to open tight junctions between epithelial cells, thus enhancing paracellular transport of peptides [79]. In addition, some studies report that chitosan-based NPs can protect insulin from enzymatic degradation in the GIT and facilitate its absorption, leading to sustained anti-hyperglycemic effects in streptozotocin-induced diabetic rat models [80]. Similarly, dextran sulfate/chitosan NPs have been reported to enhance the oral bioavailability of insulin, achieving significant glucose-lowering effects in animal studies [81]. Pectin and pectin-based composites have shown promise in colon-targeted systems, where the pectin matrix is degraded by colonic microflora, offering an additional layer of controlled release for systemic absorption [82]. Starch, a ubiquitous plant carbohydrate storage polymer found in roots, stems, tubers, and seeds [83], further exemplifies the versatility of plant PS as nanoparticle precursors. Composed of approximately 20–30% amylose and 70–80% amylopectin [84], starch can be structurally modified or assembled into nanoscale carriers with tailored degradation profiles and release kinetics. Its abundance, biocompatibility, and regulatory familiarity strengthen its appeal as a sustainable and scalable material for oral peptide delivery. Collectively, plant PS-based nanoparticles embody excellence in oral peptide formulation by uniting biocompatibility, gastrointestinal resilience, and controlled release behavior. Their capacity to protect insulin and GLP-1RAs from degradation while promoting intestinal interaction situates them as a promising and translationally relevant platform for next-generation oral peptide therapeutics.

### 4.2. Microalgae-Based Expression and Bioencapsulation for Oral Delivery

**Figure 3:**
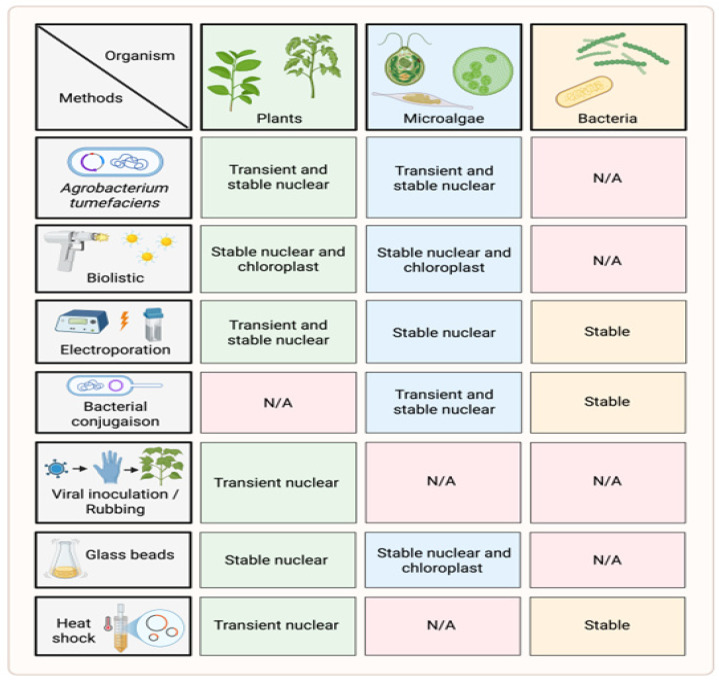
***Transient and stable transformation strategies in plants, microalgae, and bacteria*.** Various technical approaches allow stable or transient expression of heterologous peptides in plant cells, microalgae, and bacterial systems. N/A: Not applied. The figure was created in Biorender. Boscart, T. (2026) https://Biorender.com/p5l62bp.

Microalgae represent an excellent and versatile platform for the production and oral delivery of therapeutic peptides, including insulin and GLP-1RAs. These unicellular photosynthetic organisms offer rapid growth, ease of genetic manipulation, and scalable cultivation in contained photobioreactors, facilitating GMP compliance. Reported recombinant protein yields in microalgae typically range from 0.1 to 5% of total soluble protein, corresponding to approximately 10–100 mg/L culture, although higher levels have been reported for selected targets and optimized strains [93,94]. Like plants and bacteria, microalgae can be genetically engineered for stable or transient expression of heterologous peptides (Figure 3). Their unicellular nature and short doubling times enable rapid screening and production cycles, making microalgae particularly attractive for recombinant peptide development. Beyond productivity, microalgae possess intrinsic biochemical properties that favor peptide stabilization and oral delivery [95,96]. Their cells are rich in endogenous PS, which contributes to their cell walls and naturally encapsulates recombinant peptides, protecting them during the GIT [97,98]. This biopolymer matrix provides both physical shielding from proteolytic degradation and mucoadhesive properties that enhance intestinal residence time and bioavailability of orally delivered peptides [97,99].

Among microalgal platforms, *Chlamydomonas reinhardtii* is the best-characterized and most extensively used species for recombinant protein expression [100], with both nuclear and chloroplast genomes amenable to stable transformation. Targeting recombinant peptides to starch granules via the granule-bound starch synthase pathway has demonstrated enhanced protection against proteolytic degradation while preserving biological activity. Anti-malarial vaccine peptides fused to starch granules in *C. reinhardtii* elicited strong immune responses and significantly improved survival following *Plasmodium berghei* challenge in mice compared with starch-only controls[97]. Oral or intraperitoneal administration of these microalgae-produced peptides elicited strong, specific immune responses in mice and significantly improved survival after *Plasmodium berghei* challenge compared with starch-only controls [99]. Similarly, oral delivery of microalgal particles carrying heterologous antigens induced protective immunity against *Staphylococcus aureus*, further validating microalgae-based oral delivery [101].

Additional microalgae systems expand the translational potential of this approach. *Chlorella vulgaris* and *Chlorella sorokiniana* are robust species suitable for large-scale cultivation, with thick PS-rich cell walls that enhance biomolecule protection. Advances in genetic engineering have enabled recombinant protein expression in *C. vulgaris*, including chloroplast-based production of basic fibroblast growth factor, demonstrating the feasibility of producing pharmaceutically relevant proteins in this system [102]. *Dunaliella salina*, a halotolerant chlorophyte, has attracted attention for its ability to grow in hypersaline media, reducing contamination risks [103]. It has been engineered to express foreign proteins within its chloroplast, and its carotenoid-rich biomass provides additional antioxidant protection to sensitive peptides [104]. Notably, *D. salina* has recently been shown to perform protein glycosylation, with oligomannoside *N*-glycans identified as the predominant species [105].

Marine diatoms such as *Phaeodactylum tricornutum* and *Thalassiosira pseudonana* provide eukaryotic-like post-translational modifications, including complex glycosylation. They possess silica-based frustules that can function as natural microcapsules [106]. Their silica-based frustules can also act as natural microcapsules, making them attractive for peptide stabilization and targeted delivery. Recombinant expression of human functional antibodies has already been achieved in *Phaeodactylum tricornutum* [107,108,109]. In addition, thanks to the intrinsic encapsulation capacity of microalgae, recombinant peptides can be directly orally delivered by eating microalgae as nutraceuticals (Table 7). This strategy may allow therapeutic peptides such as insulin and GLP-1RAs to bypass enzymatic degradation in the GIT. Moreover, microalgae naturally synthesize a range of bioactive metabolites, including carotenoids, phenolic compounds, phycocyanins, and polyunsaturated fatty acids, which possess antioxidant, anti-inflammatory, and insulin-sensitizing properties [110]. Co-delivery of these endogenous bioactives alongside insulin or GLP-1RAs may provide synergistic metabolic benefits, potentially enhancing glycemic control and reducing oxidative stress in diabetic patients [110].

#### Microalgae-Derived Polysaccharides (Ps) Nanoparticles as Oral Delivery Carriers

Like higher plants, microalgae have emerged as a highly promising and versatile source of PS for the development of nanoparticle-based oral delivery systems. Their unique biochemical composition, extensive structural diversity, and generally recognized safety and edibility profiles position microalgae-derived PS as compelling alternatives to synthetic polymers and terrestrial plant-derived materials. Across microalgae-derived PS, key physicochemical parameters, including high molecular weight, branching complexity, charge density, and sulfation, critically govern solubility, mucoadhesion, resistance to digestive enzymes, and interactions with intestinal mucus and epithelial membranes [115]. Microalgae PS, encompass intracellular storage polysaccharides (e.g., starch-like α-glucans) and structural or extracellular polysaccharides (EPS), including sulfated galactans, rhamnans, xylans, complex heteropolysaccharides, and alginate-like polymers, all of which can be transformed into nanoparticles suitable for peptide encapsulation. Some green microalgae like *Chlamydomonas reinhardtii* also accumulate starch granules [116], which can be isolated and processed into starch-based nanoparticles enabling controlled and enzyme-responsive drug release. Species including *Chlorella*, *Arthrospira* (Spirulina), *Porphyridium*, *Dunaliella*, as well as brown microalgae, have been particularly well studied due to their high PS content, robust cultivation, and demonstrated industrial scalability [117]. Among these PS, alginate, a linear copolymer composed of β-D-mannuronic acid and α-L-guluronic acid, stands out as one of the most attractive polysaccharides for oral peptide delivery. Microalgae-derived alginate offers the potential for improved control over molecular weight, monomer composition, and batch-to-batch consistency through bioprocess engineering. Alginate’s capacity to form ionically crosslinked hydrogels and nanoparticles (e.g., Ca^2+^-alginate systems) has made it one of the most extensively validated polysaccharides for oral peptide encapsulation. In addition, alginate exhibits excellent mucoadhesive properties, pH-sensitive swelling behavior, and strong protection against gastric acidity, all of which are highly advantageous for oral delivery of labile peptides. Alginate-based delivery systems have been among the most compelling carriers studied for oral insulin and GLP-1RAs delivery (Table 8). For example, in a *db/db* mouse model of T2D, the GLP-1RA exenatide encapsulated in alginate-hyaluronate microspheres exhibited effective oral absorption, reaching a plasma concentration peak approximately 4 h post-administration [118]. This formulation produced a significant and sustained reduction in blood glucose relative to controls, achieving an estimated relative bioavailability of ~10% compared with subcutaneous injection [118]. This study highlights that alginate matrices can protect GLP-1 analog peptides from gastric degradation and enable intestinal release and uptake. For insulin, alginate and alginate-polysaccharide hybrid nanoparticles have shown significant pharmacological activity. Early work with alginate/chitosan nanoparticles in diabetic rats demonstrated that orally delivered insulin NPs could reduce basal blood glucose by more than 40% and sustain hypoglycemic responses for extended periods, with evidence of intestinal adhesion and uptake [119]. Beyond their role as passive carriers, several microalgae PS, particularly sulfated polysaccharides, exhibit intrinsic antioxidant, anti-inflammatory, and immunomodulatory activities. These bioactivities support the hypothesis that synergistic effects may be achieved with metabolic peptide therapies by improving intestinal barrier integrity and mitigating local inflammation, which are known to impair peptide absorption and efficacy [120]. Collectively, these multifunctional properties are decisive for oral peptide delivery and are particularly relevant for macromolecular therapeutics such as insulin and GLP-1 receptor agonists (GLP-1RAs), positioning microalgae-derived polysaccharides as a next-generation excipient platform for oral metabolic therapies.

## 5. Current Limitations and Technical Challenges in Plant- and Microalgae-Based Expression Platforms

**Figure 4:**
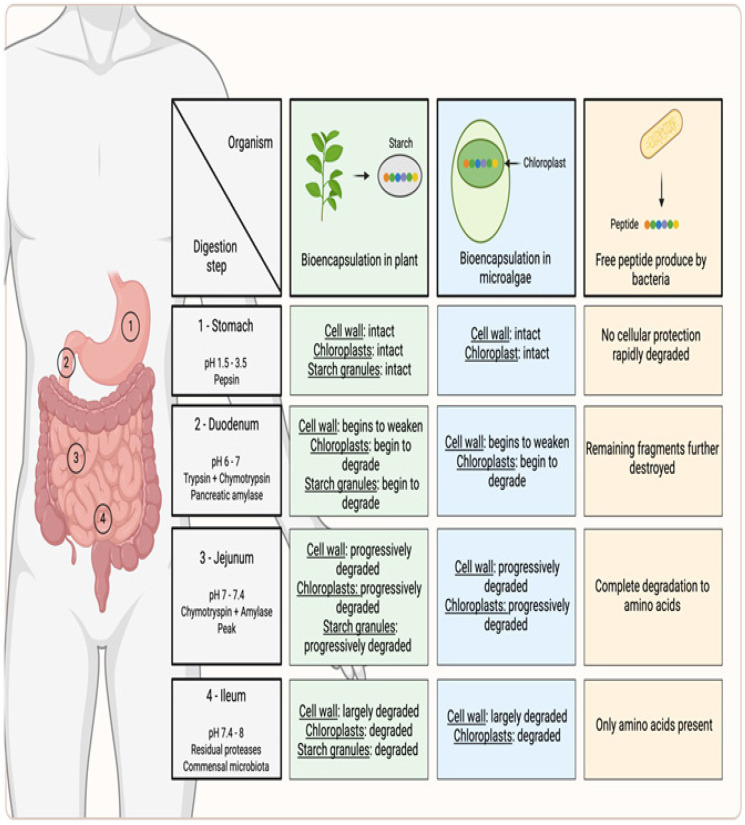
***Digestion and bioavailability of peptides in free versus bioencapsulated forms from plant and microalgae extracts*.** Predicted peptide yield during gastrointestinal transit is shown for plant- and microalgae-based bioencapsulated peptides compared with their free counterparts. Biorender. Boscart, T. (2026) https://Biorender.com/p5l62bp.

Plant- and microalgae-based expression platforms have emerged as versatile and sustainable alternatives for the production of therapeutic peptides, including insulin and GLP-1RAs. These photosynthetic organisms provide scalable, low-cost production, perform eukaryotic post-translational modifications, and allow for natural bioencapsulation strategies that protect peptides from gastrointestinal degradation, thereby enabling potential oral delivery (Figure 4). Despite these advantages, several technology-intrinsic challenges related to expression, manufacturing control, formulation stability, and regulatory affairs are still limiting their clinical translation.

### 5.1. Variability in Recombinant Peptide Expression

Recombinant peptide expression exhibits substantial variability depending on the host species, genetic engineering strategy, and cultivation conditions. Differences across biological platforms, including bacteria, yeast, mammalian cells, and photosynthetic organisms such as microalgae and higher plants, result in marked disparities in expression yield, post-translational modifications, and product consistency [124,125]. Within plant- and microalgae-based systems, the choice between nuclear and chloroplast transformation is a major determinant of expression efficiency. Chloroplast transformation often enables higher peptide accumulation due to polyploidy and reduced gene silencing as well as absence of protease degradation; however, it may limit proper folding or post-translational processing for more complex peptides [60,126]. Nuclear transformation allows for more complex modifications but is typically associated with lower and more variable expression levels. Promoter selection further contributes to variability. Constitutive promoters may drive higher baseline expression but can impose metabolic burdens on the host, whereas inducible or tissue-specific promoters introduce additional sources of heterogeneity depending on induction efficiency and developmental stage [127]. Cultivation parameters such as light intensity, nutrient availability, temperature, and growth phase also significantly influence peptide yield, particularly in microalgae and plant systems [128].

### 5.2. Batch-to-Batch Variability and Standardization Challenges

Batch-to-batch variability remains a major obstacle to clinical translation, especially when whole cells or polysaccharide-based matrices are used as delivery vehicles rather than purified peptides. Biological heterogeneity in cell populations, differences in biomass composition, and fluctuations in expression levels across production runs complicate dose standardization [129]. These challenges are amplified in orally delivered formulations based on intact cells or bioencapsulation strategies, where the active peptide is embedded within complex biological matrices. Variability in cell-wall composition, polysaccharide content, and degradation kinetics can directly affect peptide release profiles and bioavailability [130]. When PS are extracted and processed into nanoparticles, additional variability may be introduced during the extraction, purification, and formulation steps. Although microalgae-derived PS are generally considered biocompatible based on their use as food ingredients, formulation as pharmaceutical nanocarriers introduces new requirements for standardization, physicochemical characterization, and reproducibility [131].

### 5.3. Stability Issues in Oral Delivery

Peptide stability represents another significant limitation, particularly if such a peptide is intended for oral administration. Recombinant peptides are susceptible to degradation during long-term storage, with sensitivity to humidity, temperature, and oxygen leading to loss of biological activity over time [132]. Stability profiles can vary substantially between batches, especially when peptides are stored within biological matrices rather than as purified peptides. Following oral administration, additional variability arises from differences in gastrointestinal conditions, including pH, enzymatic activity, transit time, and microbiota composition. These factors can differ widely between patients and disease states, further complicating the reproducibility of therapeutic effects [133].

### 5.4. Regulatory and Manufacturing Challenges

From a regulatory perspective, plant- and microalgae-based platforms introduce complexities distinct from conventional biologics. The use of whole-cell matrices or biologically derived PS nanocarriers complicates product definition, identity testing, and quality control. Unlike purified peptide formulations, these systems rely on matrix degradation and biological processing to enable peptide release, increasing variability in product performance. Moreover, harmonized regulatory frameworks specific to biologically derived nanocarriers are still evolving. Prior food or nutraceutical use of plant or microalgae PS does not obviate the need for rigorous pharmaceutical safety assessment when these materials are used as drug delivery systems. Addressing these challenges will require standardized GMP-compliant manufacturing pipelines, robust analytical methods, and early engagement with regulatory authorities.

## 6. Translational and Clinical Relevance

Although plant- and microalgae-based oral delivery systems have demonstrated encouraging results in preclinical studies, none have yet progressed to late-stage human clinical trials. Beyond the technical constraints discussed above, clinical translation is further limited by physiological differences between preclinical models and humans, as well as by differences in platform maturity relative to established microbial systems.

### 6.1. Translational Barriers from Animal Models to Humans

One of the principal challenges in translating oral biological delivery systems from animal models to humans lies in substantial interspecies differences in gastrointestinal physiology [134,135]. Rodents, which dominate preclinical studies, exhibit shorter gastrointestinal transit times and thinner mucus layers than humans, potentially leading to overestimation of peptide stability, release efficiency, and absorption. Rodents typically possess a thinner and more rapidly renewing mucus layer compared with humans [136], facilitating closer epithelial contact and potentially enhanced absorption of peptide-loaded NPs or bioencapsulated cells. This anatomical difference may again lead to an overprediction of epithelial uptake in preclinical models, particularly for delivery systems relying on particle-mucus interactions or mucoadhesion. The gut microbiota introduces further complexity. Microbiota composition and metabolic activity vary not only between species but also among individuals, disease states, and dietary patterns [137]. Differences in digestive enzyme composition and activity further exacerbate uncertainty, as proteases critical to peptide degradation display marked interspecies variability [138,139]. Therefore, efficacy observed in animal models may not reliably predict clinical performance.

### 6.2. Platform Maturity and Clinical Readiness

From a clinical manufacturing standpoint, microbial systems such as *Escherichia coli* and yeast currently dominate clinical insulin and GLP-1RA production due to their high yields, well-established scalability, batch-to-batch reproducibility, and extensive regulatory precedent [140]. In contrast, although plant- and microalgae-based systems offer unique advantages, including a reduced risk of human pathogen contamination and intrinsic bioencapsulation, they generally exhibit lower and more variable expression yields. Delivery strategies relying on whole-cell biomass introduce additional challenges in ensuring precise dosing and controlled release, which are particularly critical for chronic metabolic therapies. Despite these hurdles, plant- and microalgae-derived PS nanoparticles remain attractive candidates for oral peptide delivery. Ongoing formulation efforts using natural polysaccharides reflect increasing interest in achieving regulatory approval for such systems [141]. With improved characterization and translational modeling, these platforms may ultimately complement existing oral biological technologies.

On the other side, early human studies of plant-derived oral vaccines demonstrate that recombinant peptides can be safely produced, bioencapsulated, and administered orally [142,143]. Building on this foundation, plant expression systems, including leafy crops such as lettuce, have been optimized to produce peptides bioencapsulated within plant cell walls, which protect sensitive molecules from gastric degradation and enable targeted intestinal release [144]. Such strategies are particularly relevant for insulin and GLP-1RAs, which are highly susceptible to proteolytic degradation in the GIT. Encapsulated microalgal systems, particularly those based on well-characterized, food-grade species, are also progressing toward translational application for insulin and GLP-1RA delivery [145]. In contrast, many nanoparticle-based formulations derived from plant or microalgal PS remain at a proof-of-concept stage, lacking comprehensive pharmacokinetic data, long-term safety evaluation, and standardized manufacturing protocols.

### 6.3. Pharmacokinetic and Pharmacodynamic Uncertainties

Significant pharmacokinetic and pharmacodynamic (PK/PD) uncertainties remain major barriers to clinical translation. Variability in peptide release, absorption, and systemic exposure can result in inconsistent therapeutic responses, particularly for peptides with narrow therapeutic windows such as insulin. Absorption kinetics are influenced by formulation disintegration, mucus penetration, epithelial transport, and first-pass metabolism, leading to poor predictability of human outcomes based on animal data. Addressing these uncertainties will require integrated PK/PD modeling, human-relevant in vitro systems, and judicious use of large-animal models to bridge the gap between preclinical promise and clinical efficacy.

## 7. Conclusions and Future Directions

Plant- and microalgae-based production platforms, combined with PS-based NPs, represent a promising frontier for the synthesis and oral delivery of insulin and GLP-1RAs. These systems offer scalable and versatile production, support correct protein folding and appropriate post-translational modifications, and inherently reduce risks associated with contamination by human pathogens or endotoxins. Their capacity to enable oral or edible formulations constitutes a major opportunity to improve patient adherence and quality of life in chronic metabolic diseases. Despite these advantages, substantial challenges remain. Translational gaps between preclinical models and human physiology, batch-to-batch variability, long-term stability, and precise dose reproducibility continue to limit clinical applicability.

From a clinical perspective, plant- and microalgae-derived oral insulin or GLP-1RA formulations are most realistically envisioned, at least in the near to medium term, as adjunct therapies rather than complete replacements for injectable formulations. In this context, oral delivery could provide low, sustained peptide exposure to complement parenteral dosing, potentially improving basal metabolic control, reducing glycemic variability, or lowering the required dose and injection frequency of injectable agents. Such adjunct strategies may be particularly attractive for GLP-1RAs, where partial receptor engagement may still yield meaningful metabolic benefits.

A second promising application lies in early-stage disease intervention or prevention, particularly in individuals with insulin resistance, prediabetes, or early T2D. In these situations, modest and more physiological peptide exposure achieved through oral bioencapsulation may be sufficient to restore metabolic homeostasis without the need for full systemic replacement therapy. Oral GLP-1RAs formulations produced in plant or microalgae platforms could also be used to reduce body weight and appetite, improve insulin sensitivity, and modulate inflammatory tone at the early stages of diseases, potentially delaying disease progression and reducing long-term treatment burden.

In contrast, the use of plant- and microalgae-derived oral formulations as full replacements for injectable insulin or GLP-1RAs remains a longer-term goal. Such an achievement will require consistent and predictable bioavailability, precise dose control, as well as robust pharmacokinetic and pharmacodynamic profiles comparable to established injectable products. Given the narrow therapeutic window of insulin and the stringent safety requirements for chronic metabolic therapies, significant technological, regulatory, and clinical hurdles must still be overcome before oral formulations can reliably replace injections in insulin-dependent patients.

Immunogenicity represents an additional and particularly important consideration. From a theoretical perspective, subtle alterations in peptide conformation, aggregation state, or post-translational modifications, as well as the presence of residual host-derived proteins or polysaccharides, could influence immune recognition. These concerns are especially relevant for chronic therapies requiring long-term exposure. However, evidence from existing plant-derived biologics provides important context. Clinical studies of plant-produced recombinant proteins, particularly oral vaccines and subunit antigens, have generally demonstrated favorable safety profiles with no unexpected systemic immune reactions [146], supporting the notion that plant-based expression systems are not intrinsically immunogenic. Similarly, food-grade microalgae and microalgae PS have a long history of human consumption, and, to date, there is only limited experimental evidence indicating clinically relevant immunogenicity attributable to microalgae expression platforms [147]. Nevertheless, the transition from nutraceutical use to pharmaceutical-grade, bioencapsulated peptide therapeutics, especially in nanoparticle formulations, introduces new variables that warrant careful investigation. While oral delivery may favor immune tolerance and reduce systemic immune activation, chronic administration of bioencapsulated insulin or GLP-1RAs requires a comprehensive evaluation of both local and systemic immune responses. Future progress in this field will therefore depend on standardized and GMP-compliant manufacturing processes, rigorous physicochemical and immunological characterization, predictive translational models, and well-designed clinical trials assessing pharmacokinetics, pharmacodynamics, safety, and immunogenicity. With continued technical optimization, regulatory alignment, and clinical validation, plant- and microalgae-based oral delivery platforms have the potential to significantly expand the therapeutic landscape for insulin, GLP-1RAs, and other peptide-based medicines, ultimately contributing to more accessible, patient-friendly, and sustainable treatments for metabolic disease.

## Figures and Tables

**Table 1:** Insulin types used in T1D and T2D.

Type	Name (Brand Name, Manufacturer)	Preparation Available	Role
Short-acting	Regular insulin (Humulin R, Lilly) (Novolin R, Novo Nordisk) Glulisine (Apidra, Sanofi-Aventis)	Vial, Pen Vial, Pen Vial, Pen	Alternative bolus insulin
Rapid-acting	Lispro (Humalog, Lilly) (Admelog, Sanofi Aventis) (Lyumjev, Lilly) Aspart (Novolog, Novo Nordisk) (Fiasp, NovoNordisk)	Vial, Cartridge, Pen Vial, Pen Vial, Pen Vial, Cartridge, Pen Vial, Cartridge, Pen	Mealtime (bolus) insulin
Intermediate-acting (insulin mixture)	NPH (Humulin N, Lilly) (Novolin N, Novonordisk) Protamine/Lispro (Humalog Mix, Lilly) Protamine/Aspart (Novolog mix, Novo Nordisk)	Vial, Pen Vial, Pen Vial, Pen Vial, Pen	Basal insulin
Long-acting	Glargine (Lantus, Sanofi-Aventis) (Toujeo, Sanofi-Aventis) (Bassaglar, Lilly) Detemir (Levemir, Novo Nordisk)	Vial, Pen, cartridge Pen Pen Vial, Pen	Basal insulin (once/twice daily)
Ultra-long-acting	Degludec (Tresiba, Novo Nordisk)	Pen	Basal insulin

**Table 2:** Types of GLP-1RAs used in T2D.

Name	Brand Name (Manufacturer)	Delivery Mode	Indication
Exenatide extended release	Bydureon (AstraZeneca)	Subcutaneous	T2D
Liraglutide	Victoza (Novo Nordisk)	Subcutaneous	T2D mainly
	Saxenda (Novo Nordisk)	Subcutaneous	Weight management
Dulaglutide	Trulicity (Lilly)	Subcutaneous	T2D
Semaglutide	Ozempic (Novo Nordisk)	Subcutaneous	T2D mainly
	Rybelsus (Novo Nordisk)	Oral	T2D
	Wegowy (Novo Nordisk)	Subcutaneous	Weight management
Tirzepatide	Mounjaro (Lilly)	Subcutaneous	Weight management

T2D: Type 2 diabetes.

**Table 3:** Advantages offered by plant-based and microalgae-based expression platforms over traditional microbial-based systems.

	Plant- and Microalgae-Based Expression Systems	Microbial-Based Systems
Cost-effectiveness	Basic inputs such as sunlight, water, and minerals significantly reduce upstream production costs.	Requires expensive fermentation infrastructure. High purification cost due to endotoxins and misfolded proteins.
Safety	Plants and microalgae are free from human pathogens and endotoxins. The risk of contamination is reduced to zero as compared to animal- and microbial-based systems.	Risk of endotoxin contamination (especially in *E. coli*). Improper folding or inclusion bodies.
Scalability	Cultivation can be scaled from greenhouses or photobioreactors to open fields or vertical farms, enabling rapid upscaling during global demand surges. Transient expression systems can also be industrialized using controlled bioreactor environments.	Scaling up bioreactors increases complexity and cost. Limited batch sizes for quality control.
Edibility and oral delivery	Therapeutic peptides can be bioencapsulated within the cells, protecting them from gastrointestinal degradation and allowing oral delivery without purification, thus reducing both cost and invasiveness.	Proteins often require extensive purification before use. Not compatible with oral delivery without further formulation.

**Table 4:** Plant-based production of recombinant protein is commercially available for research, cosmetics, and medical applications.

	Expression System	Expression Type	Application	Manufacturer
Avidin	*Zea mays* (maize)	Stable	Research	Sigma-Aldrich
β-glucuronidase				
Glucocerebrosidase (Taliglucerase—Elelyso^®^)	*Daucus carota* cells (wild carrot)		Medical treatment of Gaucher disease	Protalix Biotherapeutics/Pfizer
α-galactosidase-A (Pegunigalsidase alfa—Elfabrio^®^)	*Nicotiana tabacum*		Medical treatment of Fabry disease	Protalix Biotherapeutics/Chiesi
Growth factors	*Hordeum vulgare* (barley)		Cosmetics	ORF Genetics
Human Serum Albumin (Cellastim S^®^)	*Oryza sativa* (rice)		Research	Invitria
Human Lysozyme (OsrhLYZ)				Oryzogen/Healthgen Biotech
Human Transferrin (HyCreat, OsrhTF)				
Human Lactoferrin (OsrhLF)			Research/Cosmetics	
Human α-1 Antitrypsin (OsrhAAT)				
Human Fibronectin (OsrhFN)				
Human growth factor (OsrhEGF)				

**Table 5:** Plant-based expression systems for insulin and GLP-1RAs production.

Plant	Expression Type	Recombinant Type	References
*Nicotiana tabacum* (tobacco)	Stable	Insulin fused to CTB	[54]
*Nicotiana benthamiana*	Transient	Insulin analog SCI-57	[62]
*Lactuca sativa* (lettuce)	Stable	Insulin fused to CTB, Proinsulin	[63,64]
Transgenic *Zea mays* (maize)	Transient	Proinsulin	[65]
*Arabidopsis thaliana* (thale cress)	Stable	Insulin	[61]
*Camelina sativa* (false flax)	Stable	Insulin fused to CTB	[66]
*Nicotiana benthamiana*	Transient	Ex4	[67]
*Nicotiana tabacum*	Transient	Ex4-fused to CTB	[68]
*Lactuca sativa*	Transient	Ex4-fused to CTB	[69]
*Oryza sativa* (rice)	Stable	GLP-1 fused to GFP GLP-1 fused to globulin	[70,71]
*Transgenic tobacco* cv. 81V9	Transient	Ex4-fused to transferrin	[72]

CTB: Cholera toxin B; Ex4: Exendin-4.

**Table 6:** Plant polysaccharide-based nanoparticles for oral delivery of insulin and GLP-1RAs with antihyperglycemic effect.

Polysaccharides	Origin	Mechanism	Peptides	Formulation	References
Pectin	Apple, citrus	Protects in gastric pH; targets folate receptors in the intestine	Insulin, GLP-1	Pectin nanoparticles dual-crosslinked with calcium and adipic dihydrazide, modified with folic acid	[85,86]
Cellulose derivatives (e.g., HPMC)	Cotton, wood	Enteric coating to protect peptides	Insulin	Coating of HPMC	[87]
Arabinoxylans	Maize, rye, rice, sorghum, and wheat,	Forms viscous gels, encapsulates peptides	Insulin	enzymatic gelation of arabinoxylans, using a triaxial electrospray method	[88,89]
Guar gum	Guar beans	Sustained release matrices	Insulin	copolymer (γ-polyglutamic acid—guar gum)	[90]
Inulin	Chicory root, Jerusalem artichoke	Not digested in the upper GI; fermented in the colon. So, it enables colon-targeted release.	Insulin	Inulin-based nanoparticles or hybrid oral matrices	[91]
Starch	Corn, potato, rice,	Digestible unless chemically modified. Can be modified (e.g., cross-linked, esterified) for slow release or resistance to GI digestion, suitable for use in controlled-release oral delivery in this regard.	Insulin	Nanoparticles via nanoprecipitation or enzymatic crosslinking	[92]

**Table 7:** Microalgae-based production of recombinant protein/peptides for oral delivery.

Peptide/Protein	Microalgae Host	Delivery Format	Results	References
Green Fluorescent Protein (GFP)	*Chlamydomonas reinhardtii*	Lyophilized whole cells	GFP detected in the intestinal tissue and the bloodstream of zebrafish	[111]
Antimicrobial peptide PisL9K22WK	*Tetraselmis subcordiformis*	Fresh engineered microalgae feed	An oral antimicrobial agent significantly improved the resistance of mussels to Vibrio	[112]
Antimicrobial peptide NZ2114	*Tetraselmis subcordiformis*	microalgae feed	Turbit fed with *T. subcordiformis* transformants containing NZ2114 were resistant against *Staphylococcus aureus*, *Vibrio parahaemolyticus*, and *Vibrio splendidus*	[113]
Edible vaccine viral nervous necrosis (VNN) antigens	*Chlamydomonas reinhardtii*	microalgae feed	Oral delivery of recombinant *C. reinhardtii* producing the VNN improved immune-related gene expression and intestinal microbiota.	[114]

**Table 8:** Oral Insulin and GLP-1RA Delivery with alginate-based nanoparticles.

Alginate-Based Delivery System	Peptide	Animal Models	Key Findings	References
Alginate-hyaluronate microspheres	Exenatide	*db/db* mice	Oral exenatide reached plasma Cmax at 4 h, and normalized blood glucose	[118]
Alginate coupled to chitosan-conjugated deoxycholic acid	Insulin	Diabetic rats	Oral NPs reduced blood glucose > 40% and sustained hypoglycemia	[121]
Octaarginine-modified alginate NPs	Insulin	Diabetic rats	Enhanced intestinal uptake and controlled insulin release	[122]
Alginate microbeads containing chitosan nanoparticles	Insulin	STZ-induced diabetic mice	insulin-loaded alginate microbeads could lower blood glucose level in much prolonged period of 96 h	[123]

NPs: Nanoparticles, STZ: Streptozotocin.

## Data Availability

No datasets were generated in this study.
